# Dystocie d’expulsion sur jumeaux conjoints thoracopage à l’hôpital de base de Talangai, République du Congo

**Published:** 2011-04-25

**Authors:** Ghislain Herman N’Dinga, Léon Hervé Iloki

**Affiliations:** 1 Université Marien Ngouabi Brazzaville, Maternité Hôpital de Base de Talangaï, République du Congo; 2 Université Marien Ngouabi, Chef de Service de Gynécologie obstétrique CHU Brazzaville, République du Congo

**Keywords:** Jumeaux conjoints, Echographie, Rupture utérine, Congo

## Abstract

Les auteurs rapportent l’accouchement par voie basse des jumeaux conjoints thoracopage méconnu qui s’était soldé par l’enclavement du fœtus, première tête hors vulve, compliqué d’une rupture utérine à l’hôpital de base de Talangaï à Brazzaville, République du Congo.

## Introduction

L′accouchement à terme par voie basse des jumeaux conjoints est un événement rare pouvant mettre en jeu le pronostic maternel [1,1-4]. La nonréalisation de L′échographie par bon nombre de femmes au cours de la gestation dans notre milieu (inaccessibilité, coût élevé) expose les praticiens à une telle éventualité [5,6].

Nous rapportons un cas de dystocie d′expulsion sur jumeaux conjoints thoracopage survenue à L′hôpital de Talangaï (HBT) à Brazzaville (République du Congo).

## Patient et observation

En mars 2006 une patiente de 26 ans s’est présentée à la maternité de L′HBT pour douleurs d′accouchement au terme de 41 semaines d′aménorrhée. Elle était 3^ième^ grossesse, 2^ième^ accouchement (G_3_P_2_), avec deux enfants vivants nés par voie basse. Il n’y avait pas de notion de gémellité dans sa famille. Parturiente avait eu six consultations prénatales auprès d′une sage-femme dans un centre de santé intégré. Aucune échographie n’avait été réalisée durant toute la gestation.

A L′admission L′examen avait noté une hauteur utérine à 41cm, deux pôles céphaliques, deux foyers de bruits de cœur fœtaux (BDCF) à 140- 156 bpm, des contractions utérines régulières, environ 4/10mn, durée 30- 40 secondes, le col effacé dilaté à 8cm, membranes rompues, présentation céphalique engagée.

Devant ce tableau, L′hypothèse de grossesse gémellaire avait été évoquée et la patiente conduit en salle de naissance où après des efforts de poussée, une première tête (T1) se dégageât, et il se produisît aussitôt L′arrêt de progression du mobile fœtale.

Après échec de tant de manœuvres visant à extraire le reste du corps, L′obstétricien appelé en urgence avait posé le diagnostic d′enclavement d′un monstre double compliqué de rupture utérine devant une anémie sévère et la perception de trois mains au niveau du détroit supérieur.

La laparotomie d′urgence avait permis de mettre en évidence une rupture utérine complète latérale droite par laquelle une deuxième tête (T2) était sortie de L′utérus alors que le tronc et les membres demeuraient intra utérins retenus certainement par la première tête (T1) qui était hors vulve.

**Figure 1:**
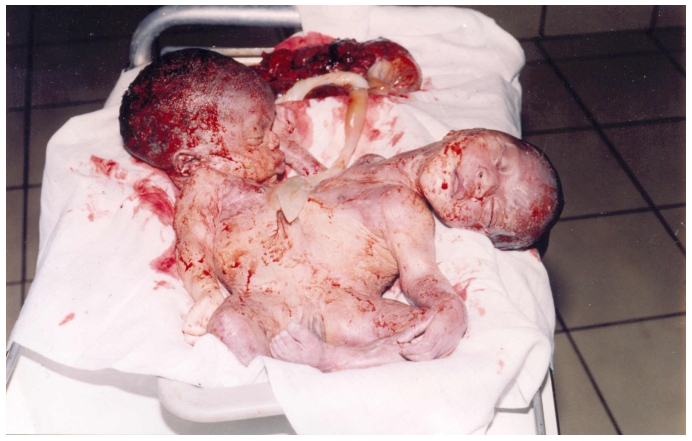
Thoracopage responsable d′une d′expulsion compliquée de rupture utérine au terme d′un suivi sans échographie chez une G3P2 de 26 ans à l′hôpital de Talangaï à Brazzaville, République du Congo

Nous avions alors pratiqué: 1) une réduction intra utérine de T1 selon la technique de Zavanelli; L′extraction par la brèche utérine d′un fœtus présentant deux têtes dont le périmètre crânien était 31cm pour T1, 33cm pour T2, deux cous, deux cages thoraciques, quatre membres supérieurs, un tronc unique, L′accolement ayant eu lieu en dessous des mamelons de L′appendice xiphoïde jusqu’à L′extrémité inférieure, un seul cordon gras, deux membres inférieurs, un anus perméable. Le sexe était ambigu. Le poids du fœtus était 4980 grammes pour une taille d′environ 51cm (à partir de T1ou de T2) (Figure 1). Le nouveau-né était en état de mort apparente, réanimé en vain. Le placenta avait pesé 730 grammes. Le cordon d′insertion central comportait deux artères et une veine centrale; 3) une hystérorraphie a été pratiquée; 4) la patiente avait reçu 4 poches de sang et du plasma frais congelé en per opératoire.

Les suites opératoires ont été simples. La patiente est sortie au 10ième jour.

## Discussion

La morphologie et la biométrie fœtale sont souvent ignorées dans notre milieu à cause du faible taux de réalisation de L′échographie au cours de la gestation [5] liée au fait que nos maternités ne disposent pas toujours d′échographe en salle de naissance dont L′importance a été démontrée [7]. Cela explique certaines situations rencontrées dans nos maternités parmi lesquelles L′accouchement de fœtus malformés à terme alors que ces grossesses auraient pu faire L′objet d′un avortement thérapeutique et éviter ainsi à la femme le risque, les dépenses et la stigmatisation encore très forte au Congo.

La fréquence des malformations congénitales est très variable, dépendant de la méthodologie de L′étude, de la région et des moyens diagnostiques. A L′hôpital de base de Talangaï à Brazzaville, Gandzien trouve une fréquence de 0,3% dans une série de 13 908 accouchements [5]. Et très souvent il s’est agi de malformations méconnues avant L′accouchement par manque d′échographie.

Les jumeaux conjoints représentent un phénomène rare. Cette rareté tient à rareté de la grossesse gémellaire, environ 1 à 2% des grossesses, à la rareté du monozygotisme, environ 35% des grossesses gémellaires, à la rareté division de L′embryon après le 14ième jour après la fécondation ou de la fusion partielle des deux lignes primitives qui conduit aux monstres doubles [1,3]. Ce cas est le tout premier accouché à L′hôpital de base de Talangaï.

Le diagnostic de jumeaux conjoints est possible avant la 12^ième^ semaine d′aménorrhée par échographie. Et lorsque qu’il n’existe aucune possibilité de les séparer, une interruption de grossesse est généralement réalisée, ceci pour prévenir les complications d′un accouchement laborieux par voie basse [1,2]. L′accouchement d′un monstre double à terme se voit donc lorsque la femme n’a pas eu d′échographie, généralement dans les pays sous développés.

En dehors des cas de prématurité, mort in utéro, faible poids où L′accouchement peut se faire par voie vaginale, généralement, c’est une césarienne qui est indiquée [2]. Dans notre cas, L′extrême enclavement avait occasionné une rupture utérine par ignorance du diagnostic.

Lorsque le diagnostic est méconnu, comme dans notre cas, L′accouchement par voie basse ou la tentative d′accouchement par voie basse peut engendrer de graves lésions maternelles et menacer ainsi le pronostic tant vital que fonctionnel [4].

La mortalité néonatale des jumeaux conjoints est généralement élevée [1]. Au Congo, la littérature locale n’a jamais décrit un cas de séparation ou de tentative de séparation des jumeaux conjoints. L′autopsie pour une étude approfondie avait été refusée par les parents.

## Conclusion

L′importance de L′échographie au cours de la grossesse et en salle de naissance impose sa généralisation et son accessibilité dans notre milieu. Ceci peut contribuer à réduire la mortalité et la morbidité maternelle encore très importantes en Afrique.

## Contribution des auteurs

Tous les auteurs ont également contribué à la rédaction du manuscrit.

## Conflits d′intérêts

Les auteurs ne déclarent aucun conflit d′intérêts
